# Significant upper urinary tract hematuria as a rare complication of high-pressure chronic retention of urine following decompression: a case report

**DOI:** 10.1186/1752-1947-6-254

**Published:** 2012-08-22

**Authors:** Ishvar Naranji, Marco Bolgeri

**Affiliations:** 1 Department of Urology, Kent and Canterbury Hospital, Ethelbert Road, Canterbury, Kent, CT1 3NG, UK

**Keywords:** Hematuria, Retention, High-pressure, Decompression

## Abstract

**Introduction:**

Hematuria has been described following bladder drainage in 2% to 16% of high-pressure chronic urinary retention treatments by decompression and is generally self-limiting. We describe a case of significant bilateral upper urinary tract hematuria following drainage of high-pressure chronic retention. To the best of our knowledge, the only similar case reported in the literature was in 1944.

**Case presentation:**

An 82-year-old Caucasian man was referred to our department with nocturnal enuresis and a palpable bladder. He was catheterized, produced a residual volume of 2900mL, and ended up becoming oliguric. Following investigations, he had bilateral nephrostomies. He was discharged 18 days after presentation.

**Conclusions:**

Clinicians should keep in mind the presentation discussed in this case report to be able to swiftly manage this extremely rare complication of decompression in patients with high-pressure chronic retention.

## Introduction

High-pressure chronic retention is maintenance of voiding, with a bladder volume of greater than 800mL and an intravesical pressure above 30cm H_2_O, accompanied by hydronephrosis [1]. The treatment involves catheterization to relieve the pressure on the kidneys and allows normalization of renal function. We anticipate hematuria in 2% to 16% of cases and a profound diuresis following drainage of the bladder [2]. We report the case of an 82-year-old Caucasian man who instead became oliguric and had significant hematuria affecting the upper renal tract.

## Case presentation

**Figure 1:**
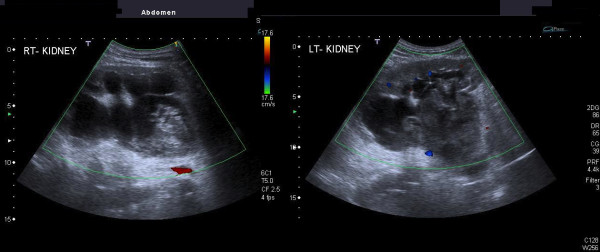
Ultrasound of renal pelvises demonstrating bilateral hydronephrosis and echogenic material.

An 82-year-old Caucasian man was referred by his general practitioner because of nocturnal enuresis, declining renal function, and a palpable bladder. He was taking aspirin for ischemic heart disease. On admission, his estimated glomerular filtration rate (eGFR) was 12mL/minute (creatinine was 398mmol/L), hemoglobin was 9.5g/dL, platelet count was 357 (×10^9^/L), and coagulation profile was normal. Insertion of a urethral catheter produced a residual volume of 2900mL. He was put on intravenous fluid replacement and monitored for diuresis. Over the following 48 hours, he developed significant hematuria causing anemia (hemoglobin of 7.8g/dL) and requiring bladder irrigation and blood transfusion. He became oliguric and his renal function deteriorated to an eGFR of 7mL/minute (creatinine of 670mmol/L). An ultrasound evaluation revealed an enlarged prostate (150mL) and bilateral hydronephrosis with echogenic material within the pelvicalyceal systems (Figure 1).

**Figure 2:**
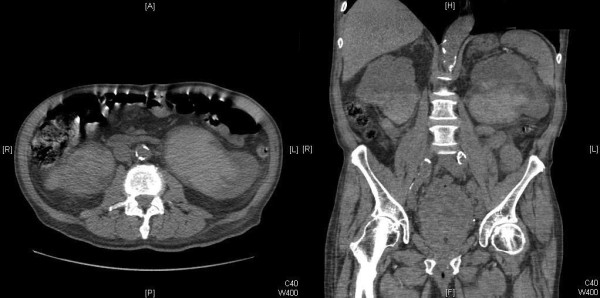
Computed tomography scan confirming ultrasound findings.

A computed tomography scan confirmed dilatation of both renal pelvises and both ureters filled with material consistent with blood clots (Figure 2).

**Figure 3:**
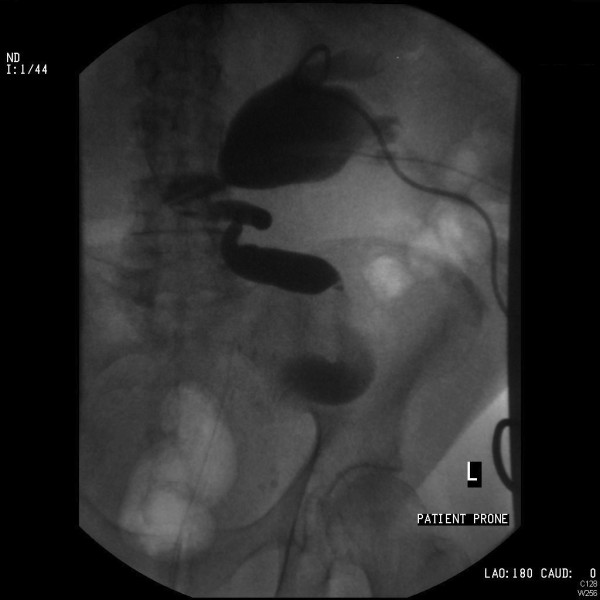
Left nephrostogram demonstrating resolution of clots and a dilated tortuous ureter.

Our patient developed leukocytosis (20,000/mm^3^) with a C-reactive protein of 188mg/L and so bilateral 8-French nephrostomies were inserted on day 4. Despite regular flushing, they became blocked on day 6. Cystoscopy revealed a large occlusive prostate and a trabeculated bladder with numerous diverticulae. After an attempt at bilateral retrograde stenting was unsuccessful because of dilated and very tortuous ureters filled with clots, he underwent percutaneous nephroscopic examination and washout of the left renal pelvis and insertion of a 24-French drain. No stones or malignancies were identified. He declined the procedure on the right side, where the nephrostomy was left *in situ* and was flushed regularly until an accidental dislodgement. His urine output increased significantly, reflecting a progressive improvement in renal function. The hematuria eventually settled. Repeat ultrasound and left nephrostogram demonstrated a clearance of the clots from the collecting system (Figure 3).

The left nephrostomy was removed, and our patient was discharged on day 18 with an indwelling urethral catheter and listed for an urgent transurethral resection of the prostate. His eGFR at discharge was 26mL/minute.

## Discussion

The treatment of high-pressure chronic urinary retention is decompression via a urinary catheter. Hematuria has been described following bladder drainage in 2% to 16% [2] of cases of urinary retention and generally is a self-limiting event. Gradual decompression of the bladder has not been shown to decrease the occurrence of hematuria, possibly because the damage to the bladder wall precedes catheterization [3]. In the absence of randomized controlled trials, the literature supports quick and complete emptying of the obstructed bladder in all cases of retention [2]. Significant hematuria can occur in a number of cases after operative relief of ureteric obstruction but this can be attributed to the surgical trauma rather than to the effect of decompression on the urothelium. We described a case of significant bilateral upper urinary tract hematuria following drainage of high-pressure chronic retention. The only similar case reported in the literature [4] involved a 69-year-old man who developed frank hematuria and fatal uremia following bladder decompression. Cystoscopy revealed blood draining from both ureteric orifices, and a post-mortem examination showed bilaterally distended upper tracts filled with blood clots.

## Conclusions

Significant hematuria affecting the upper urinary tract and leading to obstructive renal failure is an extremely rare complication of decompression in patients with high-pressure chronic retention. Perhaps a lesser degree of hematuria often goes unrecognized and is wrongly attributed to surgical trauma or bladder origin. In the case of worsening renal function after catheterization and the presence of hematuria, this possibility should be considered.

## Consent

Written informed consent was obtained from the patient for publication of this case report and any accompanying images. A copy of the written consent is available for review by the Editor-in-Chief of this journal.

## Abbreviation

eGFR, Estimated glomerular filtration rate.

## Competing interests

The authors declare that they have no competing interests.

## Authors’ contributions

IN and MB wrote the manuscript and performed data collection. MB reviewed the literature and revised the manuscript. Both authors read and approved the final manuscript.
